# Supplementary material for: MetaRibo-Seq measures translation in microbiomes
Source: Nat Commun. 2020 Jun 29;11:3268. doi: 10.1038/s41467-020-17081-z (PMC7324362; doi:10.1038/s41467-020-17081-z)
Supplement: Supplementary file 10 — Supplementary Data 7 [file 41467_2020_17081_MOESM10_ESM.zip › File2/Confidence_VeryHigh_Taxonomy/321329_out.krona.html]

Javascript must be enabled to view this page.

members
magnitude
magnitudeUnassigned
count
unassigned
taxon
rank

321329\_out

7

superkingdom
2
7

phylum
7
976

class
7
200643

7
171549
order

815
7
family

genus
7
816


SRS014235\_contig\_number\_40529SRS015579\_contig\_number\_contig-100\_259.150428SRS019685\_contig\_number\_34775SRS019787\_contig\_number\_13106SRS053398\_contig\_number\_contig-100\_157.163291SRS075341\_contig\_number\_21661SRS1055034\_contig\_number\_11795
species
7
246787
